# Paracetamol versus ibuprofen in treating episodic tension-type headache: a systematic review and network meta-analysis

**DOI:** 10.1038/s41598-023-48910-y

**Published:** 2023-12-06

**Authors:** Ammar Alnasser, Hassan Alhumrran, Mustafa Alfehaid, Mustafa Alhamoud, Nada Albunaian, Mazen Ferwana

**Affiliations:** 1 Family Medicine Department, Eastern Health Cluster, Ministry of Health, Eastern Province, Saudi Arabia; 2 Family Medicine Academy, Eastern Health Cluster, Ministry of Health, Eastern Province, Saudi Arabia; 3 KAMC, MNGHA, KSAU-HS, Riyad, Saudi Arabia

**Keywords:** Neurological disorders, Headache

## Abstract

Tension-type headache (TTH) is the most common type of headache worldwide. It is defined and classified according to the International Classification of Headache Disorders. TTH is treated with over-the-counter medications, mostly paracetamol or ibuprofen. The purpose was to assess the effectiveness of paracetamol versus ibuprofen in treating episodic tension-type headache (ETTH) through direct and indirect comparisons of randomized controlled trials (RCTs). We included RCTs comparing paracetamol with a placebo, ibuprofen with a placebo, or paracetamol with ibuprofen for acute ETTH treatment that were published between 1988 and 2022. We searched the Cochrane Central Register of Controlled Trials (CENTRAL), MEDLINE, EMBASE, and the Web of Science. The Cochrane Collaboration risk of bias tool was used to assess the risk of bias. We identified 14 studies including 6521 people with ETTH. None of the studies had a low risk of bias for all domains; this was most likely due to inadequate reporting and a small sample size. Ibuprofen (odds ratio (OR): 1.73, 95% confidence interval (CI): 1.17–2.56) showed better efficacy than paracetamol (OR: 1.62, 95% CI 1.24–2.13) for pain-free status at 2 h, while paracetamol (OR: 1.42, 95% CI 0.87–2.30) showed better efficacy than ibuprofen (OR: 1.20, 95% CI 0.58–2.48) for pain-free status at 1 h. Paracetamol was associated with the lowest likelihood of rescue medication use (OR: 0.49, 95% CI 0.37–0.65). Ibuprofen was associated with a lower likelihood of the occurrence of any events and gastrointestinal adverse events compared with placebo and paracetamol (OR: 0.95, 95% CI 0.64–1.41 and OR: 0.81, 95% CI 0.44–1.50, respectively). Paracetamol and ibuprofen showed better efficacy than placebo in treating ETTH; there was no statistically significant difference in efficacy between the two drugs. For individuals at a higher risk (like renal insufficiency or risk of GI bleeding), paracetamol may be considered as a preferred option instead of Ibuprofen. Further meta-analyses of head-to-head trials are needed for direct comparisons in the future.

*PROSPERO registration number*: CRD42022340936.

## Introduction

Tension-type headache (TTH) is a type of primary headache as defined by the International Classification of Headache Disorders (ICHD) classification^1^, which was first published in 1988 and updated in 2004 (ICHD-2) and 2018 (ICHD-3), without significant differences. TTH is classified as infrequent episodic, frequent episodic, and chronic TTH^1^.

The general prevalence of headaches in a person's lifetime is over 90%^2^. The global prevalence of TTH is 26.1% in the community, affecting 1.89 billion people, making it the most common type of headache worldwide and the third most prevalent disorder, with higher prevalence in females than in males^3–5^. Stress and mental tension are reported to be the most common precipitating factors^6^. TTH impacts socioeconomic status through medical services cost and sick leave^1,3–5,7^.

TTH is usually self-treated without medical advice, which unfortunately leads to suboptimal management^8,9^. There are different modalities of treatment, including nonpharmacological (such as relaxation therapy and cognitive therapy) and pharmacological (such as nonsteroidal anti-inflammatory drugs (NSAIDs) and paracetamol) treatments^10^. Several societies worldwide have tried to standardize the treatment of TTH through their clinical guidelines^9,11–14^. Most published guidelines suggest treating episodic tension-type headache (ETTH) with NSAIDs, paracetamol, aspirin + paracetamol + caffeine, or paracetamol + caffeine.

NSAIDs (especially ibuprofen) and paracetamol are recommended as first-line treatments^9,11,13,14^, with NSAIDs being more effective^11,13,14^. One guideline recommended combination therapy, including ibuprofen or diclofenac as first-line therapy and paracetamol as second-line therapy^12^.

As the guidelines and recommendations from multiple societies attempt to define best practices for treating ETTH, there are still issues regarding the quality and methodology of randomized control trials (RCTs)^15^. There is a paucity of RCTs with a direct comparison between paracetamol and ibuprofen.

Three RCTs directly compared paracetamol with ibuprofen in the treatment of ETTH^16–18^. The first study showed a limited effect of ibuprofen 400 mg compared with paracetamol 1000 mg and placebo. This effect was described as exploratory due to underenrollment and early termination of the study for many reasons^16^. The second study concluded that paracetamol and ibuprofen are well tolerated and significantly more effective than placebo in symptom relief, with a more significant effect of ibuprofen than paracetamol. This study applied the Ad Hoc Committee on Classification of Headache diagnostic criteria for ETTH for enrolled participants, and the study's primary outcome was not specified^17^. The third study used first perceptible pain relief and meaningful pain relief as outcomes and showed a significantly earlier time to relief with ibuprofen use than paracetamol use^18^.

Several studies compared paracetamol with a placebo in the treatment of ETTH, and many of them showed significant superiority of paracetamol 1000 mg regarding efficacy and tolerance^8,19–24^. Some studies showed no significant difference^25,26^. On the other hand, several studies compared ibuprofen with a placebo, and many of them showed significant superiority of ibuprofen 400 mg regarding efficacy^27–30^ and tolerability^27,28^.

There were two Cochrane reviews and non-Cochrane reviews in the literature. Verhagen et al. included 41 RCTs in their meta-analysis and concluded that NSAIDs were more effective than placebo, with ibuprofen having a favorable side effect profile; paracetamol was considered an alternative. Most of the included studies were published before 1995 and no specific diagnostic criteria were used^31^. Manzano, Doyon-Trottier, and Bailey found limited evidence in the literature to support the superiority of ibuprofen over paracetamol in benign headache management (including TTH) in children and adults. No specific diagnostic criteria were used^32^. A low dose of NSAIDs showed a statistically insignificant difference compared with paracetamol in the meta-analysis by Yoon et al.^33^. They suggested that high doses of NSAIDs may provide more analgesic effects than paracetamol but cause more side effects^33^. In another comprehensive review of ETTH oral treatment, paracetamol, ibuprofen, and ketoprofen were found to be more effective than placebo, with a high number needed to treat (NNT). No conclusive evidence supports any agent's superiority over other agents^34^. In the two Cochrane reviews conducted in 2015 and 2016, ibuprofen and paracetamol were significantly superior to placebo in the pain-free 2-h outcome ^15,35^. A direct comparison between paracetamol and ibuprofen was performed in a limited number of studies (3 studies), which showed no difference between these two medications regarding pain-free status at 2 h. Based on very low-quality evidence, there was a significant difference in pain-free status at 4 h in favor of ibuprofen^15^.

To the authors' knowledge, the last systematic review was conducted to address ETTH management with these medications in 2015 and 2016 and updated in 2019 without additional studies or changes in outcomes. More RCTs are needed to directly compare the effectiveness of paracetamol and ibuprofen in treating ETTH, which will enable a preliminary conclusion to be drawn from the previous systematic reviews and guidelines. For this reason, we decided to conduct a network meta-analysis to indirectly compare the effectiveness of these medications, which is the first review conducted using network meta-analysis in the ETTH treatment field.

The study aimed to assess the effectiveness difference between paracetamol and ibuprofen in treating ETTH. This aim was achieved by developing a search strategy, screening for relevant RCTs, assessing the eligibility criteria, and directly and indirectly comparing RCTs.

## Methods

### Literature search

A systematic review was conducted through title and abstract screening, including RCTs published in all languages from 1988 to 1 June 2022 with human subjects, using the following databases: Cochrane Central Register of Controlled Trials (CENTRAL) (via Ovid), EMBASE (via Ovid), MEDLINE (via Clarivate) and Web of Science (via Clarivate).

A search strategy using relevant keywords (see Supplementary note online) was performed by different search modalities, such as medical subject headings (MeSH) and text words using the Boolean operators OR for synonyms of the same concept and AND for combining different concepts. Additionally, we manually searched the reference lists of previous systematic reviews and the included trials for additional studies.

### Selection criteria

#### Types of studies

We included RCTs published from 1988 to 2022, including parallel, crossover, and double-blinded trials, comparing paracetamol with placebo, ibuprofen with placebo, or paracetamol with ibuprofen in the treatment of acute ETTH, regardless of study language or study setting.

We excluded studies without available full text, abstracts only, reviews, and studies without data of interest.

#### Participants

Study participants included adults (18 years old and older), individuals of both sexes, individuals who met the ICHD criteria for ETTH diagnosis, and individuals who did not have psychiatric disorders that require treatment, significant cognitive disorders, or other significant chronic pain disorders.

We excluded studies with participants with chronic TTH or other headaches, such as migraine.

#### Types of intervention

All included studies had at least one arm that used oral paracetamol (1000 mg), ibuprofen (400 mg) or either of them compared with a placebo for acute ETTH treatment.

#### Types of outcomes

The primary outcome was a pain-free status at two hours using any standard pain assessment method and without rescue medication use.

The secondary outcomes were a pain-free status at one hour, the use of rescue medication, and the occurrence of any adverse event and gastrointestinal (GI) adverse events.

### Selection of relevant studies:

Two independent authors (A.Y.N. and M.S.F.) reviewed the titles and abstracts to exclude irrelevant articles. The full-text assessment was performed to determine the eligible articles based on the inclusion criteria. Disagreement between the two reviewers was resolved through a discussion with a third author (N.A.B.).

### Assessment of methodological quality and risk of bias

As described in the Cochrane Handbook, two independent authors (M.A.H. and H.A.H.) assessed the included studies for risk of bias (RoB). The method used to generate the randomization sequence, allocation concealment, the determination of whether blinding was implemented for participants or staff, and whether there was evidence of selective reporting of the outcomes was recorded. It was judged that 'yes' indicated a low risk of bias, while 'no' indicated a high risk of bias for each item. Subjects were allowed to select 'insufficient' if a judgment could not be made. Review Manager version 5.3.3 was used to generate the RoB table (see Figs. 3 and 4). There was a rereview of the articles and a discussion with a third author (M.S.F.) for any disagreements.

### Data abstraction

Two independent authors (A.Y.N. and M.S.F.) abstracted the study design, number and characteristics of participants, medications and their doses, baseline headache intensity, and study outcomes. If a disagreement occurred, a discussion with a third author (N.A.B.) was applied to resolve it.

### Statistical analysis

Effect sizes for the network meta-analysis were described with 95% confidence intervals (CIs). Statistical validity was guaranteed when the 95% CI did not include 1.

The efficacy of intervention medications and placebo was measured by calculating the odds ratio (OR) with a 95% CI from the original articles. We used RevMan 5.4^36^ for pairwise meta-analysis and netmetaXL V1.6 for winbug1.4.3^37^ to perform network meta-analysis.

A funnel plot was not created due to the insufficient number of studies.

## Results

### Identification of relevant studies

**Figure 1:**
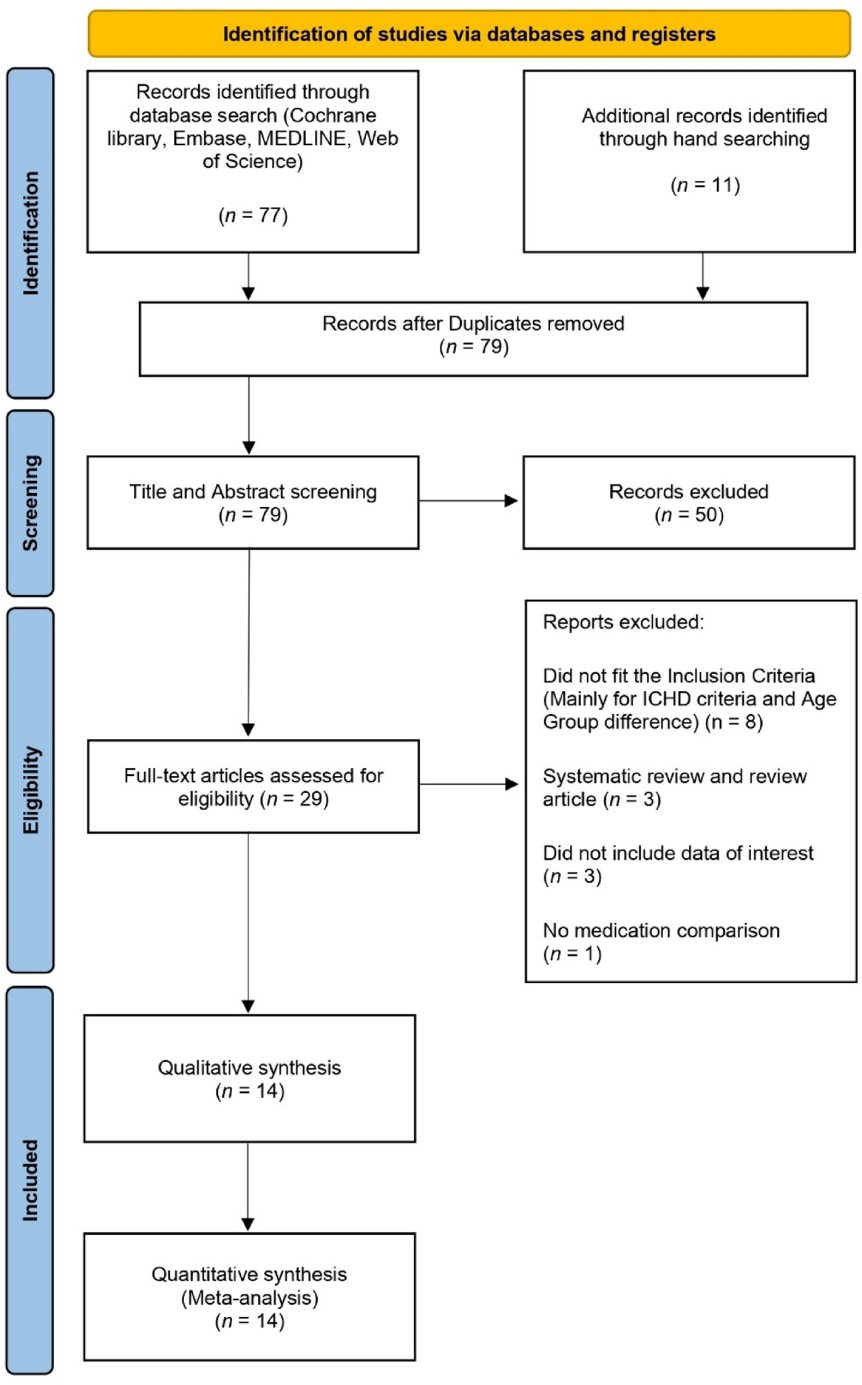
PRISMA flow diagram.

The flow diagram shown in Fig. 1 shows how relevant studies were identified. A total of eighty-eight studies were identified by searching four primary databases (Cochrane Library, Embase, MEDLINE, and Web of Science) and by hand searching. After removing duplicates and screening titles and abstracts, we obtained twenty-nine articles to be assessed for eligibility. Among these articles, fifteen were excluded from the final analysis. The following articles were excluded during the final review: articles that did not fit the inclusion criteria, articles that mainly followed the ICHD criteria and had an age group difference (n = 8), review articles (n = 3), articles that did not include data of interest (n = 3), and articles that did not perform a medication comparison (n = 1). The final 14 studies were entered into the meta-analysis.

### Characteristics of the studies included in the final analysis

In 14 RCTs (parallel and crossover), we identified a total of 6521 participants (2472 received placebo, 3097 received paracetamol, and 952 received ibuprofen), all of whom were adults with ETTH defined by IHS diagnostic criteria (ICHD). The included studies were published between 1988 and 2022. One article reported two studies with different outcomes and methodologies; the first was a crossover study, and the second was a parallel study^38^. Another article included six studies in one report; the first four studies were pooled together, and the last two were pooled together^39^. The average headache intensity at baseline in all studies was moderate to severe. One study directly compared paracetamol and ibuprofen in the treatment of ETTH^38^. Six studies compared paracetamol with a placebo, and the other six compared ibuprofen with a placebo. Table 1 shows the clinical data of the included studies.

**Table 1:** Clinical data of included studies.

Study (Year of publication)	Methodology	Participants						Intervention (dose)		headache intensity At Baseline
	Study Design	Total # of patients	# of patient ( mean age )			Gender in % ( F = Female )				
			Paracetamol	Ibuprofen	Placebo	Paracetamol	Ibuprofen	Paracetamol	Ibuprofen	
1-Diamond 2001	Randomized controlled trial, Parallel study	385	–	99 (37)	48 (36)	–	F: 79%	–	400 mg	Severe in four points scale (3)
2-Jayawardena 2014	Randomized controlled trial	891	-	Standard IBU: 342 (30.6) IBU Na = 362 (30.6)	187 (29.7)	–	Standard IBU: F: 59% IBU Na: F : 58.6%	–	Standard IBU = 400 mg IBU Na = 512 mg	Moderately severe vs Severe in the headache studies
3-Kubitzek 2002	Randomized, Parallel clinical trial	684	–	151 (45)	153 (40)	–	F: 61.6%	–	400 mg	52.3% moderate in four points scale (2). 47.7% severe in four points scale (3)
4-Prior 2002	Randomized, Parallel-group study	915	304 (33)	–	301 (34)	F : 78%	–	1000 mg	–	Moderate intensity 0–4 scale (2)
5-Yong Yue 2017	2 Studies: 1- Randomized, four-way Crossover Study 2- Randomized controlled trial, parallel group study	Studies: 1- 66 2- 157	Study 1: 49 (42) Study 2:-	Study 1: 51 (42) Study 2: 62 (38.1)	Study 1: 50 (42) Study 2: 33 (38.5)	Study 1: F: 66.7% Study 2: -	Study 1: F: 66.7% Study 2: F: 71%	Study 1: 1000 mg Study 2: -	Study 1: 400 mg Study 2: 400 mg	Study1- moderate vs severe using eDiary on 5 points categorical scale. Study 2- moderate vs severe using eDiary on 4 points categorical scale
6-Diener 2005	Randomized controlled trial, Parallel group study	1983	251 (39)	–	128 (37)	F: 73%	–	1000 mg	–	30 mm at baseline – 100 mm Visual analog scale
7- Packman 2015	Randomized, Parallel group study	226	–	IBUMOT = 89 (44.8) IBUNa = 91(42.3)	46 (39.9)	–	IBUMOT: F: 66.3% IBUNa: F: 65.9%	–	IBUMOT: 400 mg IBUNa: 512 mg	At least moderately severe – using 4 points Categorical pain severity rating and 100 mm Visual analog scale (score > 2 on categorical PSR and confirmed by > 66 mm on VAS)
8-Steiner 1998	Randomized , Parallel group study	348	123 (39)	–	116 (42)	F: 76%	–	1000 mg	–	Mild to Moderate – 60mm using VAS
9-Gerven 1996	Randomized , Parallel group study	166	–	41 (38.8)	39 (39.1)	–	F : 27%	–	200 mg	Moderate using VAS score of 25% or more at baseline
10-dahlof 1996	Randomized, five-period, cross-over study	40	40 (45)	–	40 (45)	F : 67.5%	–	500 mg and 1000 mg	–	Moderate or severe more than 55mm—VAS
11-migliardi 1994	6 Randomized, two-period crossover study	4 studies: 1900 2 studies:911	4 studies total: 1376 2 studies total: 669(33)	–	4 studies total: 689 2 studies total: 332 (33)	The 4 studies: 1st study: F = 79% 2nd study: F = 81% 3rd study: F = 77%4th study: F = 93% The 2 studies: 1st study: F = 82% 2nd study: F = 83%	–	1000 mg	–	At least moderate intensity, 4-point ordinal scale
12-mehlisch 1998	Randomized controlled trial, Parallel group study	737 randomized, 703 took medication (631 in analysis)	174 (166 included) (32)	–	172(151 included) (32)	F : 71%	–	1000 mg	–	88% Moderate intensity and 12% severe – 4 points scale
13-steiner 2003	Randomized, Parallel group study	542	- PAR 500 mg: 105 (39.7)- PAR 1000 mg: 111 (38.4)	–	112 (40.6)	F = 71%	–	500 mg and 1000 mg	–	Moderate intensity at baseline VAS > 57 mm
14-Laveneziana 1996	Randomized, cross over	30	–	26 (—)	26 (—)	–	–	–	Ibuprofen arginine No data	60 mm on VAS

**Figure 2:**
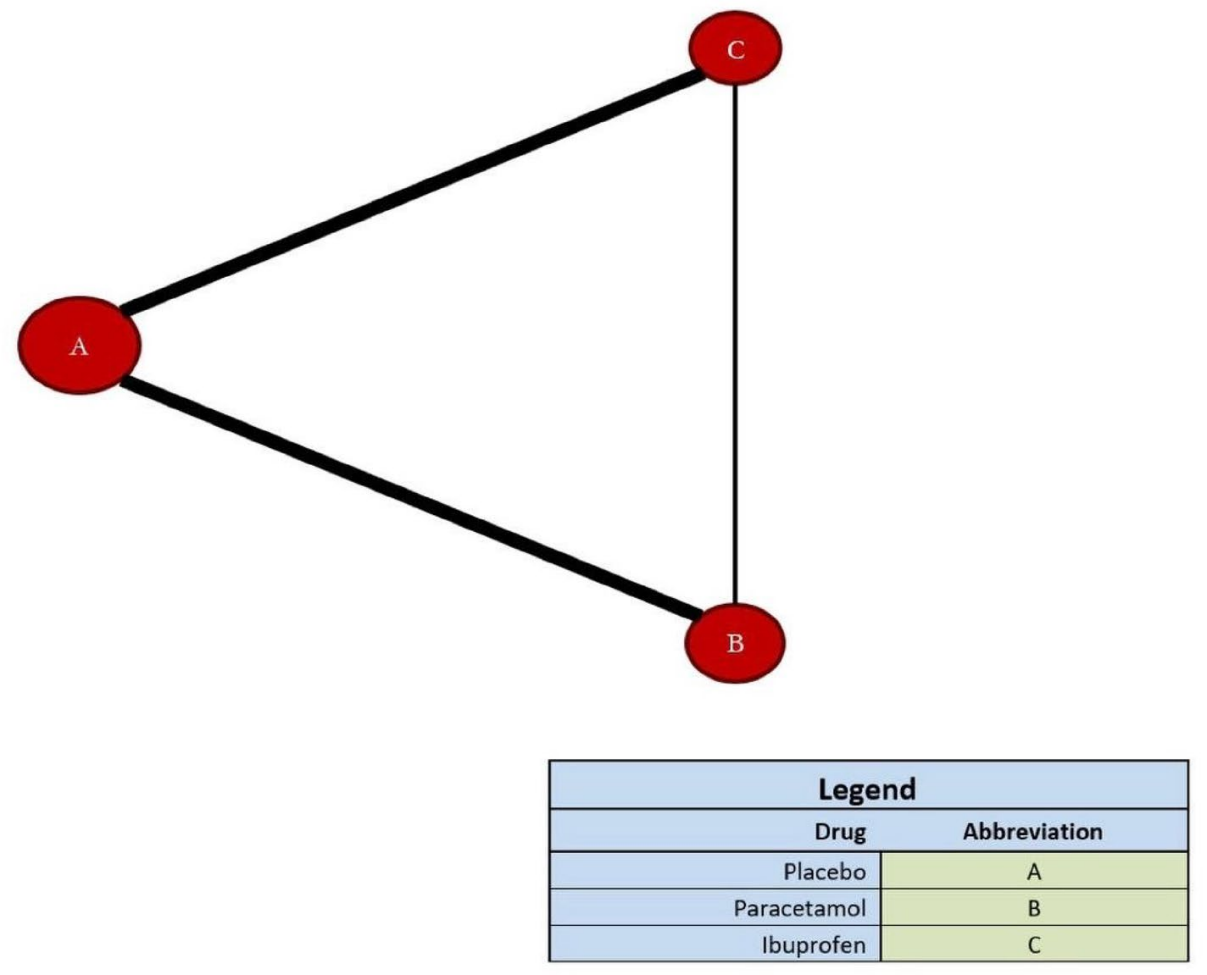
Network plot for relevant studies.

Figure 2 shows the network plot of relevant studies. Circles represent each drug as a node, and lines represent direct comparisons. The extent of the circle indicates the number of included participants receiving each drug, and the line thickness indicates the number of studies included in each comparison. Placebo had the largest node.

### Methodological quality

**Figure 3:**
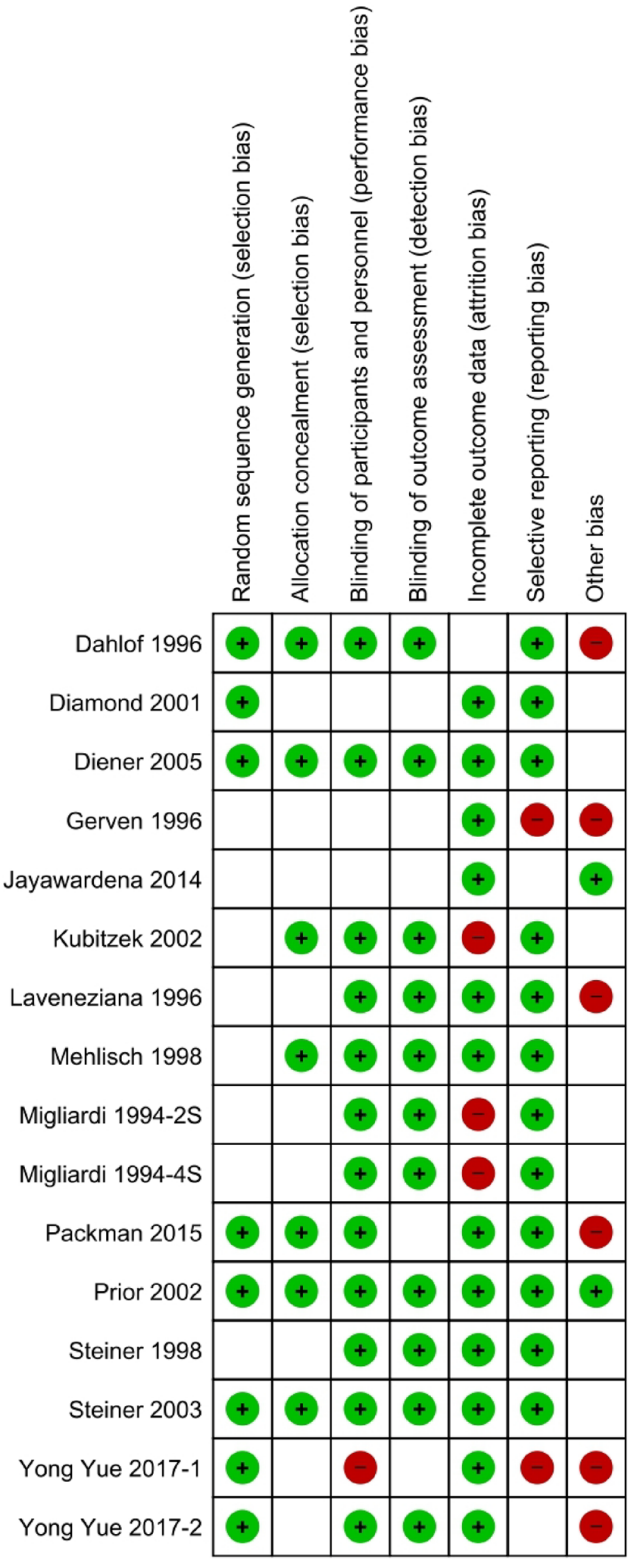
Risk of bias table.

**Figure 4:**
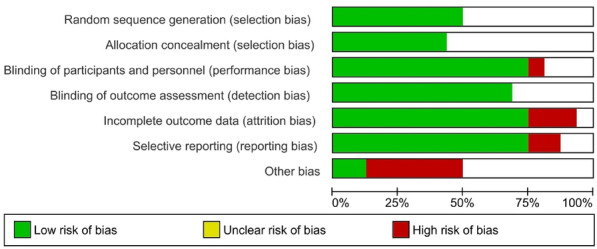
Summary of risk of bias.

**Figure 5:**
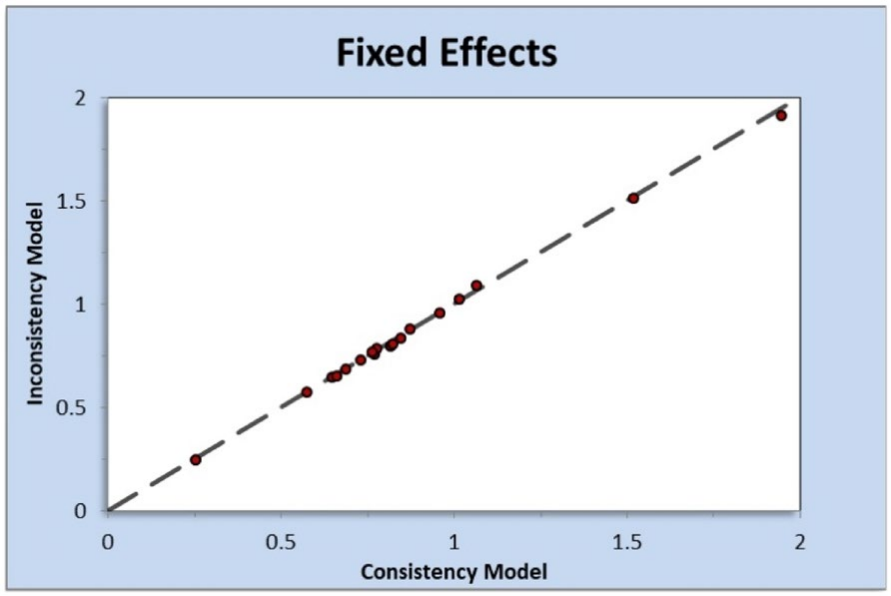
Inconsistency plot of enrolled studies (pain free at 2 h outcome).

For the methodological quality of the included studies, half of the studies had a low risk of bias in random sequence generation, and double blinding was not consistent in all the included studies. Only three studies had a high risk of attrition bias, and another two had a high risk of reporting bias. Most other articles had a low RoB. Details of the quality characteristics of each study are demonstrated in Fig. 3, which provided a summary of the RoB. Overall, studies had a low RoB, as shown in Fig. 4. The inconsistency plot of the included studies showed a fixed effect (Fig. 5).

### Comparative efficacy of paracetamol, ibuprofen, and placebo

**Figure 6:**
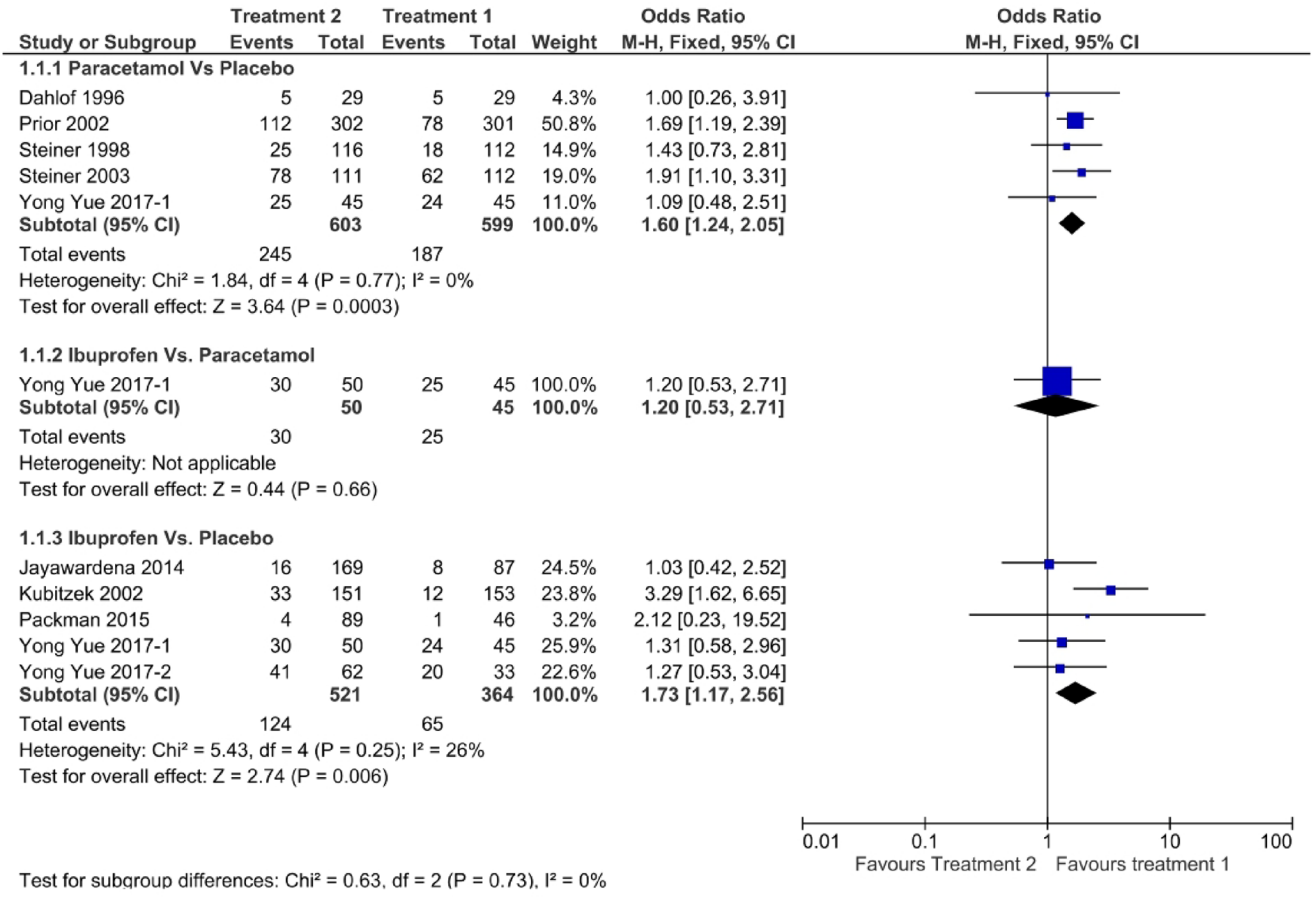
Forest plot of pain free at 2 h.

**Figure 7:**
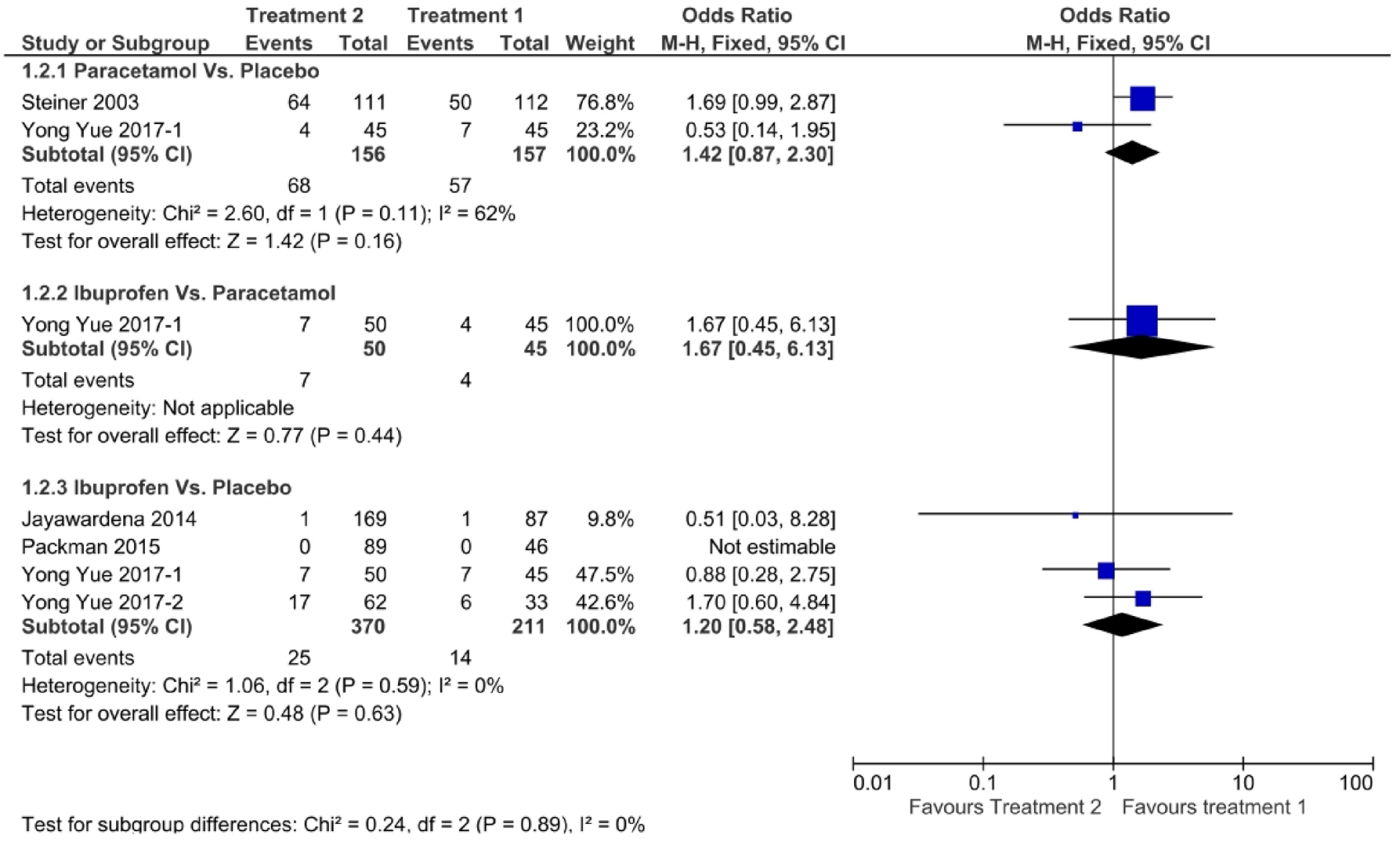
Forest plot of pain free at 1 h.

**Figure 8:**
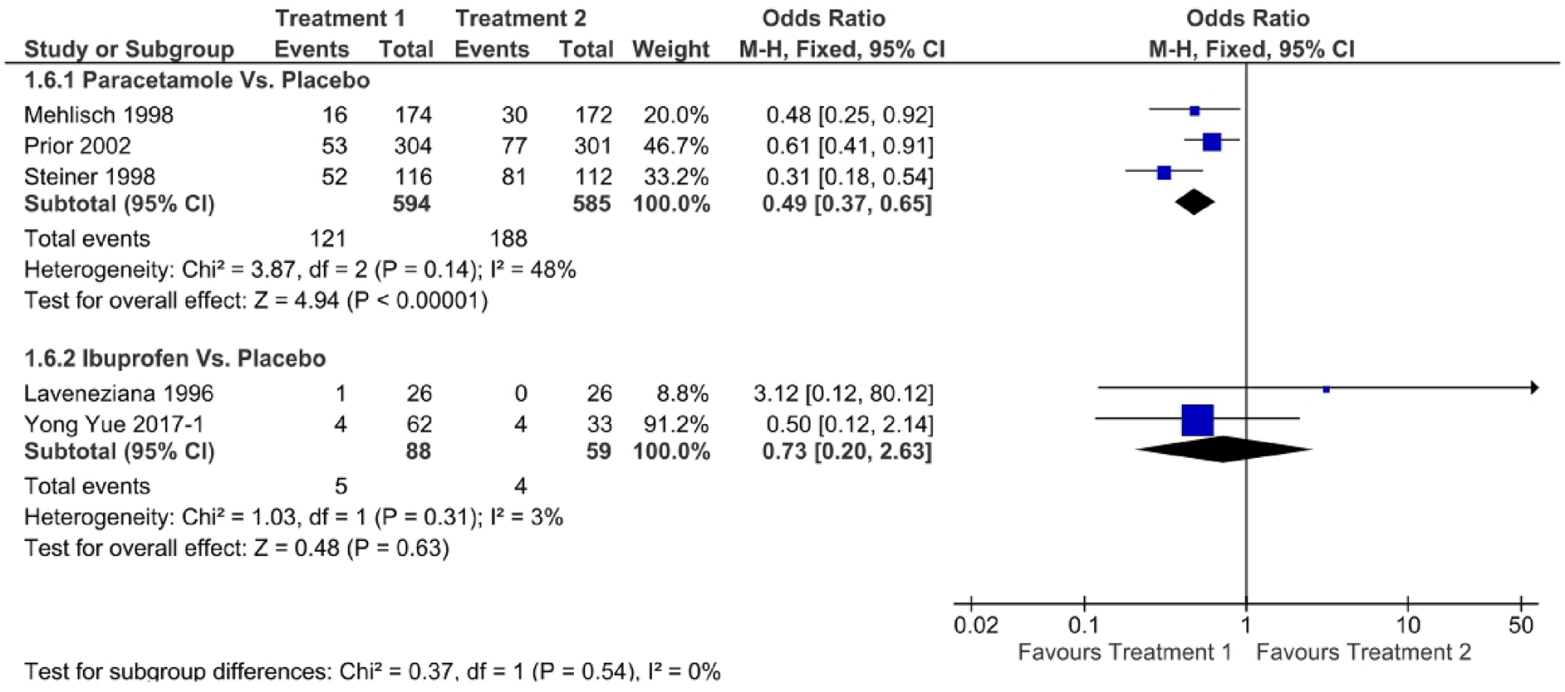
Forest plot of rescue medication used.

Figure 6 shows a forest plot of pain-free status at 2 h. The relative efficacy is plotted as the OR with the 95% CI. Ibuprofen (OR: 1.73, 95% CI 1.17–2.56) showed better efficacy than paracetamol (OR: 1.62, 95% CI 1.24–2.13). Paracetamol (OR: 1.42, 95% CI 0.87–2.30) showed better efficacy than ibuprofen (OR: 1.20, 95% CI 0.58–2.48) in pain-free status at 1 h, as shown in Fig. 7. One study directly compared paracetamol and ibuprofen, and the difference was not statistically significant (*P* = 0.66). The forest plot in Fig. 8 shows data on the use of rescue medication. Paracetamol was associated with the lowest likelihood of rescue medication use compared with ibuprofen and placebo (OR: 0.49, 95% CI 0.37–0.65).

### Adverse events related to the studied medications:

**Figure 9:**
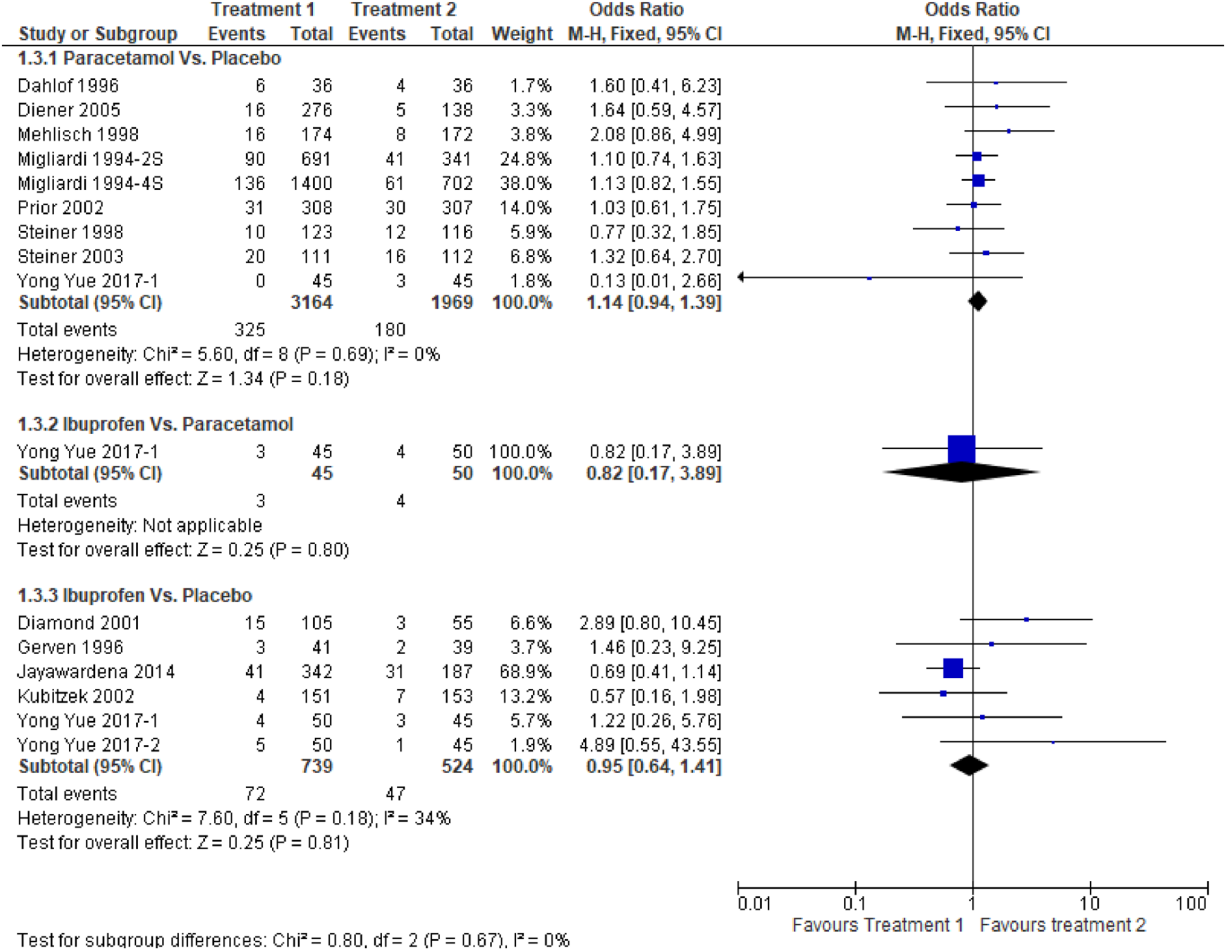
Forest plot of any adverse events.

**Figure 10:**
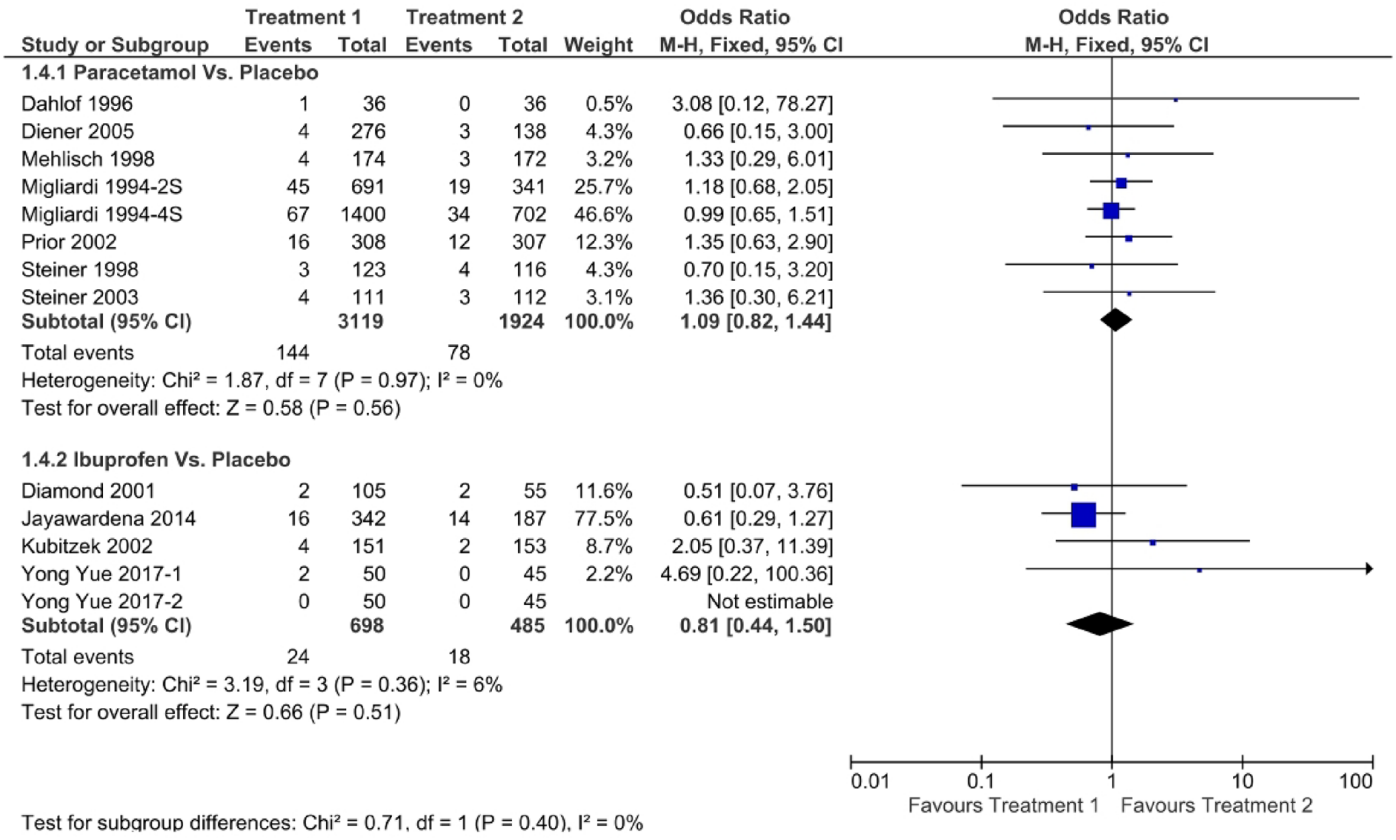
Forest plot of GI adverse events.

A variety of adverse events related to the studied medications included any adverse events or GI adverse events. A network meta-analysis was conducted for the occurrence of any adverse events, as shown in Fig. 9, and GI adverse events, as shown in Fig. 10. There was no statistical difference of any adverse and GI adverse events of ibuprofen compared with placebo and paracetamol (OR: 0.95, 95% CI 0.64–1.41 and OR: 0.81, 95% CI 0.44–1.50, respectively). Among all reported adverse events, all of them were considered a mild and no major adverse event were reported. The most reported adverse events for paracetamol are stomach discomfort (112/325) and dizziness (40/325) while the most adverse events related to ibuprofen are nausea (11/72) and dizziness (9/72).

## Discussion

Our study aimed to assess the effectiveness difference between paracetamol and ibuprofen in treating ETTH through direct and indirect RCTs.

There was no statistically significant difference between paracetamol and ibuprofen in pain-free status at 1 and 2 h. There was no heterogeneity. It is difficult to conclude which medication is more effective regarding pain-free status at 1 or 2 h. Ibuprofen showed a better effect on pain-free status at 2 h, while paracetamol showed a better effect on pain-free status at 1 h. Participants taking paracetamol showed less rescue medication use than those taking ibuprofen; this difference was not statistically significant for paracetamol, with no heterogeneity.

Regarding any adverse events, all studies reported mild side effects, including GI adverse events. Analysis of any adverse event and GI adverse events showed a statistically insignificant difference between paracetamol and ibuprofen.

In this review, the decision was made to perform network meta-analysis, even though it has lower quality than pairwise meta-analysis, to overcome the paucity in direct comparisons between paracetamol and ibuprofen based on our search. The ICHD definition, first published in 1988, was chosen because it is widely accepted and the leading definition worldwide. Nevertheless, there were no significant changes regarding the criteria until 2022^1^.

Many of our outcomes agree with several trials and reviews. Paracetamol and ibuprofen are significantly superior to placebo regarding pain-free status at two hours^15,17,31,34,35^ but not pain-free status at one hour^15,35,38^. Regarding the head-to-head comparison, a Cochrane review^15^ showed that there was nonstatistically significant superiority of ibuprofen regarding pain-free status at two hours, and the one-hour outcome was not analyzed due to the small number of events. This conclusion was based on one published trial and two nonpublished trials, with reported apparent heterogeneity between them. In the Cochrane review, the trials included applied IHS diagnostic criteria and other diagnostic criteria. A recent trial^38^ also found nonstatistically significant superiority of ibuprofen regarding pain-free status at two hours. This result was inconclusive because the study stopped before reaching the planned number of subjects for enrollment due to business and enrollment issues.

Schachtel et al.^17^ showed better significant efficacy of ibuprofen regarding pain-free status at two hours, but at one hour, there was a limited number of events that could not be analyzed. This trial is the only trial found to be titled as a direct head-to-head comparison, and they used Ad hoc diagnostic criteria in their inclusion criteria. Another trial^18^ reported statistically significant superiority of ibuprofen but used different outcomes, such as first perceptive pain relief and meaningful pain relief.

EFNS and BASH guidelines recommend ibuprofen as the drug of choice in ETTH treatment and describe paracetamol as less effective. Both guidelines were not based on systematic reviews. The Danish and Canadian guidelines recommend ibuprofen or paracetamol as first-line therapy; they depended on EFNS guidelines in their recommendation.

Paracetamol was favored regarding pain-free status at 1 h and had the lowest likelihood of rescue medication use, but the difference was statistically insignificant. However, a direct study (Yong Yue's 2017–1) favored ibuprofen regarding one-hour pain-free status, but the difference was statistically insignificant.

Regarding the use of rescue medications, our result agrees with the Cochrane review that paracetamol is significantly superior to a placebo. When paracetamol was compared with ibuprofen, it showed insignificant superiority, as the included studies demonstrated the use of rescue medications after 2 h; this result may be associated with the superiority of paracetamol in pain-free status at 1 h.

Regarding all adverse or GI events, the results of the review are not consistent with other reports. Literature shows that ibuprofen has favorable GI adverse events compared with other NSAIDs, but it is not favorable over paracetamol, neither are statistically significant^31,40^. This inconsistency could be by chance, methodological pitfalls, or other unknown reasons. For individuals with a risk of GI bleeding or using anticoagulants, paracetamol may be preferred over ibuprofen with a caution of liver injury that may associated with a large amount of paracetamol^31,40^.

Nonetheless, there are some limitations. There was only one study that performed a direct comparison between paracetamol and ibuprofen. Another area for improvement is related to the quality of the included studies, most of which had one or more forms of bias. In addition, a relatively small number of studies was found, and some lacked data of interest. To overcome this issue, the decision was made to include all parallel and crossover RCTs. Additionally, we excluded three studies with missing data; the authors were contacted but did not respond. Regarding excluded studies, we excluded three because they constituted an unethical alteration of the risk—benefit relationship. Additionally, we searched for ongoing studies, and no study was found.

## Conclusion

Paracetamol and ibuprofen showed better efficacy than placebo in treating ETTH; there was no statistically significant difference in efficacy between the two drugs. For individuals at a higher risk (like renal insufficiency or risk of GI bleeding), paracetamol may be considered as a preferred option instead of Ibuprofen. Further meta-analyses of head-to-head trials are needed for direct comparisons in the future.

### Supplementary Information

Supplementary Information.

## Data Availability

All data generated or analyzed during this study are included in this published article and its Supplementary File.

## Abbreviations

BASH: British Association for the Study of Headache
CI: Confidence interval
EFNS: European Federation of Neurological Societies
ETTH: Episodic tension-type headache
GI: Gastrointestinal
IBU: Ibuprofen
ICHD: International Classification of Headache Disorders
IHS: International Headache Society
NMA: Network meta-analysis
NNT: Number needed to treat
NSAID: Nonsteroidal anti-inflammatory drug
OR: Odds ratio
OTC: Over-the-counter
PAR: Paracetamol
RCT: Randomized controlled trial
RoB: Risk of bias
TTH: Tension-type headache
