# Supplementary material for: Paracetamol versus ibuprofen in treating episodic tension-type headache: a systematic review and network meta-analysis
Source: Sci Rep. 2023 Dec 6;13:21532. doi: 10.1038/s41598-023-48910-y (PMC10700436; doi:10.1038/s41598-023-48910-y)
Supplement: Supplementary file 1 — Supplementary Information. [file 41598_2023_48910_MOESM1_ESM.docx]

**Supplementary note: Search strategy**

1. Search keywords Phrase 1:

(acetaminophen OR 4 acetamidophenol OR 4 acetaminophenol OR 4 acetylaminophenol OR 4 hydroxyacetanilide OR 4' hydroxyacetanilide OR acenol OR acephen OR acetamino phenol OR acetaminophen OR acetaminophenol OR acetylaminophenol OR apamide OR apap OR apotel OR benuron OR calpol OR dafalgan OR doliprane OR duorol OR efferalgan OR fendon OR fibrinol OR gelocatil OR n acetyl 4 aminophenol OR n acetyl para aminophenol OR napap OR nebs OR nevral OR pamol OR panadol OR panadol soluble OR panasorb OR panodil OR paracetamole OR paralen OR parama OR perfalgan OR phenaphen OR prompt OR sedes a OR tachipirina OR tempra OR tramil OR treuphadol OR tylenol OR valadol OR datril)

1. Search keywords Phrase 2:

(ibuprofen OR Brufen OR Advil OR Motrin OR Nurofen OR Isobutylphenyl propionic acid)

1. Search keywords Phrase 3:

(Tension Headache OR Muscle Contraction Headache OR Ordinary Headache OR Stress Headache OR Essential Headache OR

Tension-Type Headache OR Headache, Tension-Type OR Headaches, Tension-Type OR Tension Type Headache OR Tension-Type Headaches OR Idiopathic Headache OR Headache, Idiopathic OR Headaches, Idiopathic OR Idiopathic Headaches OR Stress Headache OR Headache, Stress OR Headaches, Stress OR Stress Headaches OR Tension Headache OR Headache, Tension OR Headaches, Tension OR Tension Headaches OR Psychogenic Headache OR Headache, Psychogenic OR Headaches, Psychogenic OR Psychogenic Headaches OR Psychomyogenic Headache OR Psychomyogenic Headaches OR Tension-Vascular Headache OR Headache, Tension-Vascular OR Headaches, Tension-Vascular OR Tension Vascular Headache OR Tension-Vascular Headaches)

1. Search keywords Phrase 4:

(randomized controlled trial OR controlled clinical trial OR randomized controlled trials OR random allocation OR randomly OR double-blind method OR single-blind method OR clinical trial OR clinical trials)

1. #1 AND #2 AND #3 AND #4 (from 1988-2022)
